# Bioactive Compounds in Garlic (*Allium sativum*) and Black Garlic as Antigout Agents, Using Computer Simulation

**DOI:** 10.3390/life12081131

**Published:** 2022-07-27

**Authors:** Ayu Rahmania Lestari, Irmanida Batubara, Setyanto Tri Wahyudi, Auliya Ilmiawati, Suminar Setiati Achmadi

**Affiliations:** 1 Department of Chemistry, IPB University, Kampus IPB Dramaga, Bogor 16680, Indonesia; ayurl12898@gmail.com (A.R.L.); aulia_ilmiawati@apps.ipb.ac.id (A.I.); ssachmadi@apps.ipb.ac.id (S.S.A.); 2 Tropical Biopharmaca Research Center, Institute of Research and Community Services, IPB University, Bogor 16128, Indonesia; stwahyudi@apps.ipb.ac.id; 3 Department of Physics, IPB University, Kampus IPB Dramaga, Bogor 16680, Indonesia

**Keywords:** drug discovery, mass spectrum, LC-MS/MS, compound–protein interaction, black garlic, in silico, metabolomics, PLS-DA

## Abstract

Uric acid, which causes gout, is the end product of purine catabolism, synthesized by xanthine oxidase, guanine deaminase, adenine deaminase, purine nucleoside phosphorylase, and 5-nucleotidase II. Garlic contains bioactive compounds that have potential as antigout agents. Garlic fermentation to black garlic changes its components, which may affect its beneficial potential. This study aimed to select types of garlic (Indonesian garlic) and imported garlic, and to predict the interaction between their compounds and five target proteins through an in silico approach and a multivariate analysis, namely partial least squares-discriminant analysis (PLS-DA), to determine their different constituents. The target proteins were collected from open-access databases, and the compounds were identified using mass spectrometry data. The PLS-DA score plot succeeded in classifying the samples into three classes, with each class having a discriminatory compound. Based on the in silico studies, we predicted the best binding score of the five target proteins with seven important compounds: alliin, N-acetyl-S-allyl-L-cysteine, ajoene, pyridoxal, pyridoxamine, 4-guanidinobutyric acid, and D-glucosamine. These were mostly found in black garlic, with no different concentrations in the local and imported samples. Through this approach, we concluded that black garlic is a better candidate for antigout treatments, as several compounds were found to have good binding to the target proteins.

## 1. Introduction

Gout is a purine metabolism disorder characterized by increased uric acid levels in the blood (hyperuricemia). This increase causes the deposition of monosodium urate (MSU) crystals in the joints and other tissues, which can cause arthritis [1]. Gout is associated with various health complications. International observational studies have shown a strong relationship between gout and comorbidities such as chronic kidney disease, cardiovascular disease, hypertension, and depression [2]. Subsequent studies have also demonstrated that gout is associated with an increased risk of death, mainly due to cardiovascular disease [3]. The incidence of gout worldwide is gradually increasing, due to poor dietary habits, consumption of fast food, lack of physical activity, increased incidence of obesity, and metabolic syndrome [4]. Therefore, the production of uric acid in the blood needs to be reduced by inhibiting various enzymes involved in the production of uric acid. Purines are mainly degraded by the enzyme xanthine oxidase (XO) [5], which develops hyperuricemia. Besides xanthine oxidase (XO), there is also xanthine dehydrogenase (XDH). XDH can be reversibly converted to xanthine oxidase (XO) by oxidation of cysteine residues or irreversibly by limited proteolysis. XO utilizes hypoxanthine or xanthine as a substrate and O_2_ as a cofactor to produce superoxide (O_2_^−^) and uric acid. XDH acts on these same substrates but utilizes NAD as a cofactor to produce NADH instead of ·O_2_^−^ and uric acid [6]. Apart from inhibiting the action of the XO enzyme, this can also be achieved by inhibiting adenine deaminase (ADA), guanine deaminase (GDA) [7], purine nucleoside phosphorylase (PNP) [8], and 5-nucleotidase II (NT5C2) [9]. The clinical treatment for gout sufferers is to give drugs such as allopurinol and febuxostat, but these drugs have side effects, such as kidney toxicity, liver necrosis, and allergic reactions [10]. For this reason, it is necessary to search for better alternatives based on the use of medicinal plants, because of their availability, easy accessibility, and harmlessness [11].

The use of spices as herbal medicines has become a trend globally [12]. One of the spices used as a herbal medicine is garlic (*Allium sativum* L.), belonging to the genus *Allium*. Garlic is usually used as a kitchen spice; in addition, garlic has other health benefits, such as curing cardiovascular disease and other diseases [13]. Garlic can be fermented into black garlic, and during the fermentation process, the chemical compounds in garlic change and the color of the garlic changes to black; therefore, it is called black garlic. Black garlic has a sweeter taste and a less pungent aroma. Black garlic comes from Japan and has become popular in several countries such as China, South Korea, the United States, and Europe [14]. Changes in physicochemical properties due to fermentation are the main reason for the increased bioactivity of black garlic compared to garlic [15]. Therefore, besides being consumed as a spice in cooking, black garlic is also used as a medicine. Garlic and black garlic bulbs contain various active compounds, such as organosulfur compounds, flavonoids, alkaloids, polyphenols, and phenolics, that play a role in biological activities, such as being antioxidant, anti-inflammatory, anticancer, and antithrombotic [16,17]. The antigout ability of the bioactive compounds in garlic and black garlic is has not been widely reported. Research from Johnson et al. (2018) [18] reported that the *S*-allyl-L-cysteine compound found in garlic bulbs was able to reduce uric acid levels in the body through inhibition of the xanthine oxidase enzyme. Therefore, it is necessary to explore the content of compounds in garlic and black garlic as antigout candidates.

In this study, a computer simulation was used in the Autodock Vina program for initial screening, to determine the compounds found in garlic and black garlic as antigout candidates. The computer simulation approach we used was ensemble-based docking. The reason for choosing ensemble-based docking was because proteins are dynamic molecules and understanding their movement, especially with regard to protein–ligand interactions, is a significant challenge for structure-based drug discovery. In most cases, protein flexibility is under-represented in computer-aided drug design, due to uncertainty about how it should be accurately modeled, as well as the computational costs associated with incorporating flexibility in calculations. One approach that aims to address this problem is ensemble-based docking. With this technique, the ligands are anchored to an ensemble of rigid protein conformations [19]. Before the in silico process was carried out, we first identified the profile of compounds contained in garlic and black garlic extracts from both local (from Indonesia) and imported garlic, using an LC-MS/MS instrument. We also used a metabolomics approach, namely partial least squares-discriminant analysis (PLS-DA) multivariate analysis, to determine the grouping pattern of compounds in garlic by type (garlic and black garlic) and source (local and imported) and to determine discriminator compounds in each sample.

## 2. Materials and Methods

### 2.1. Preparation and Sample Extraction

The samples used were both local and imported garlic and black garlic. Local garlic from Karanganyar, Central Java, Indonesia, and imported garlic from China. The black garlic fermentation process was carried out using a black garlic fermenting machine (Shanghai Iven Pharmatech Engineering Co., Ltd., New Jinqiao Road, Pudong District, Shanghai, China) with a temperature of 65–85 °C, with controlled humidity (60–90%) for 20 days.

The extraction was carried out by maceration with methanol pa from Merck (Darmstadt, Germany) as a solvent, with a ratio of material:solvent of 1:10 for 24 h. After that, the mixture was sonicated at 55 °C for 30 min, and filtered to obtain a liquid extract. The liquid extract was then evaporated with a rotary evaporator (175 mbar at 40 for 2–3 h) to obtain a thick extract. All extracts were stored at 4 °C. Then, the solution was filtered using a 0.2 µm PTFE filter membrane. Extracts were made with three replications.

### 2.2. Identification of Metabolites by UHPLC-Q-Orbitrap-MS/MS

Preparation and analysis with UHPLC-Q-Orbitrap-MS/MS referred to the method of Emir et al. (2020) and Emir and Emir (2021) with slight modifications [20,21]. Twelve local and imported garlic and black garlic extracts were identified using UHPLC-Q-Orbitrap-MS/MS for their metabolites. Garlic and black garlic metabolites were separated using a Vanquish Flex UHPLC-Q-Orbitrap HRMS (Thermo Fisher Scientific, Waltham, MA, USA) with an *accucore* ^TM^ C18 column, 100 × 2.1 mm, and 1.5 µm (Thermo Fisher Scientific, Waltham, MA, USA). A gradient elution system with a flow rate of 0.2 mL/min for 50 min was used to separate the metabolites. A total of 0.5 µL of the filtrate was injected into UHPLC-Q-Orbitrap-MS/MS. The composition of the mobile phase used was 0.1% formic acid in water (A) and 0.1% formic acid in acetonitrile (B), with a gradient elution system of 5–35% B (0–4 min), 35–65% B (4–7 min), 65–80% B (7–15 min), 80–95% B (15–20 min), 95% B (20 min), and 95–5% B (22–22.010 min). The ionization mode used was positive Electrospray Ionization (ESI), and Q-Orbitrap was used as a mass analyzer. The ionization energies used were 18, 35, and 53 eV. The m/z range was 100–1500 in MS and MS/MS modes. Other parameters used were resolving power 70,000 FWHM, capillary temperature 320 °C, column temperature 30 °C, spray voltage (+) 3.8 Kv, and sheath and auxiliary gas 15 and 3 mL/minute. The type of scan used was full MS/dd MS2.

The metabolites were identified using mass spectra obtained from UHPLC-Q-Orbitrap-MS/MS and processed using Compound Discoverer version 2.3. We used the Mass Spectrophotometry Data Center (National Institute of Standards and Technology, Waltham, MA, USA). Identification of metabolites through selected spectral steps equalized retention times, detected unknown compounds, classified unknown compounds, predicted composition, searched for mass lists, filled gaps, and normalized areas [22].

### 2.3. Statistical Analysis

The percent abundance value of each compound was analyzed for variance with a completely randomized design using SPSS 25. Multiple Duncan’s tests were also used to determine the significance value between groups. This analysis tested whether or not there was a significant effect on the percentage abundance of each compound in each sample.

Multivariate data analysis was carried out using data obtained from the LC-MS/MS results, in the form of the peak area of each identified compound. The multivariate data analysis technique used was pattern recognition with supervision, namely partial least squares discriminant analysis (PLS-DA) with SIMCA^®^ software (v.17.0.2 Sartorius-Umetric, Umeå, Sweden). Soft Independent Modelling by Class Analogy (SIMCA^®^) is a statistical method for supervised classification of data. Model validation was carried out through permutation and cross-validation tests (CV-ANOVA).

### 2.4. Molecular Dynamics

#### 2.4.1. Protein Preparation

The target proteins xanthine oxidase (XO) (PDB ID: 2E1Q), adenine deaminase (ADA) (PDB ID: 3IAR), guanine deaminase (GDA) (PDB ID: 4AQL), purine nucleoside phosphorylase (PNP) (PDB ID: 1RSZ), and 5-nucleotidase II (NT5C2) (PDB ID: 2JC9) were obtained from the PDB protein database (https://www.rcsb.org/) (accessed on 4 January 2022) in 3D with the PDB (Protein Data Bank) format. Proteins were processed using pdb4amber to remove water and hydrogen. Subsequently, the pKa value of the ionized group in the amino acid residue was calculated using the H++ website (http://biophysics.cs.vt.edu/) (accessed on 10 January 2022) at pH 7.4. This file was then used for protein preparation with the pdbamber script. The file was parameterized by tLEap using the amber force field ff19SB. Parameters that had to be adjusted included the explicit solvent box topology using cubic, the water model using TIP3P, and neutralized using Na^+^/Cl^−^ [23].

#### 2.4.2. Molecular Dynamics Simulation

Molecular dynamics simulations were carried out on AMBER20, to provide an overview of the stability of the protein. This was carried out in four stages: minimization, heating, equilibration, and production. The minimization step was divided into five stages, with the first four stages using restraint. Each minimization stage consisted of 1000 cycles, with the first 50 cycles using the steepest descent and the remainder using the conjugate gradient algorithm. The minimization result was used as the initial input for the heating stage. The heating process was carried out with the canonical ensemble (NVT) (substance (N), volume (V), and temperature (T) are conserved). The heated protein was then equilibrated using the NVT and isothermal–isobaric (NPT) (substance (N), pressure (P), and temperature (T) are conserved) ensembles. The first equilibration stage used an NVT ensemble and the second equilibration stage used the NPT ensemble. Finally, the system was heated to a temperature of 310 K. The last stage was a production simulation carried out for 100 ns using Particle Mesh Ewald Molecular Dynamin (PMED.CUDA). Data analysis included root mean square deviation (RMSD).

### 2.5. Ensemble Docking

Proteins from the molecular dynamics simulations carried out for 100 ns were captured every 10 ns, so that ten confirmations were obtained for each protein. Each conformation was prepared using pdb4amber, by removing the hydrogen atom and changing the amino acids HID, HIP, and HIE to HIS. The prepared conformations were downloaded in pdb format, then each conformation was aligned with the X-ray structure of the protein using PyMOL, and hydrogen atoms were added. The conformation files were saved in pdb format and docked using Autodock4. The docking stage prepared the ligands and receptors. The ligands used in this study were discriminator compounds from multivariate analysis, PLS-DA, as well as reference ligands (11 original substrates and 5 commercial compounds) for each receptor (Supplementary Materials, Table S1). The ligands were obtained from the PubChem Compound database in SDF (structured data file) format in 3D, then Orca2 geometry optimization was carried out to resemble their natural state. Next, ligand preparation was carried out using AutodockTools 1.5.6. Beginning by adding a hydrogen atom, detecting the root, setting the number of torsions, and choosing torsions. The ligand file was saved as pdbqt (Protein Data Bank (PDB), Partial Charge (Q), and Atom Type (T)) format.

Next, a grid box was made with dimensions of *x*, *y*, and *z,* of 60 × 60 × 60, with a spacing of 0.5 Å. Gridbox size data were stored in a gpf file. Docking parameters were selected, the genetic algorithm (GA) was selected, with a GA run of 200, population size of 300, and the maximum number of evals selected as long (25 million). Genetic algorithm (GA) parameter data were stored in a dpf file. The binding of the tested ligand and receptor was carried out in two stages: first running auto grid, and second running autodock4 [24]. The analyses of the docking results, in the form of Gibbs free energy (∆*G*) and visualization of residue interactions (amino acids) with protein, were observed using Ligplus and ChimeraX 1.3. The binding pose was chosen based on the energy affinity (∆G) and the lowest inhibition constant (KI) with the highest number of clusters.

### 2.6. Screening of Bioactive Compounds as Drug Candidates

All compounds targeted by drug candidates were subjected to Lipinski rule of five testing and ADMET (absorption, distribution, metabolism, excretion, and toxicity) testing through the ADMET website (https://admet.scbdd.com/home/index/) (accessed on 22 March 2022). Bioavaibility, human intestinal absorption (HIA), AMES mutagenesis, carcinogenicity, and LD_50_ were used as ADMTE descriptors.

## 3. Results

### 3.1. Metabolite Profiles of Garlic and Black Garlic Extracts

**Figure 1:**
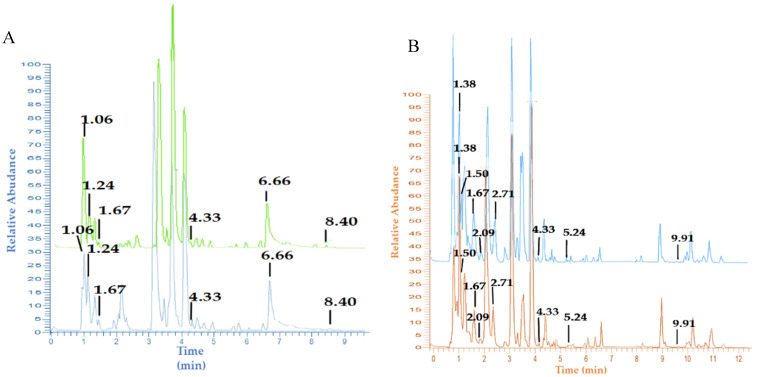
LC-MS/MS chromatogram of garlic extract (**A**) and black garlic extract (**B**); Local garlic extract (green), imported garlic (blue), local black garlic (light blue), imported black garlic (orange).

The identification of the profile of chemical compounds contained in both local (Indonesia) and imported garlic and black garlic extracts in this study utilized a non-targeted approach. The chromatogram data showed that local (Karanganyar, Central Java, Indonesia) and imported (China) garlic had the same separation pattern, but differed in their peak intensity (Figure 1A); while black garlic, both local and imported, had the same separation pattern and different peak intensities (Figure 1B). The same separation pattern with different peak intensities showed that the distribution of metabolites in each sample was almost the same, but they differed in their concentrations. The pattern of peaks with the same retention time indicated the same compound.

Garlic has a separation pattern (Figure 1A) and produces several peaks up to a retention time of 9 min, while black garlic has a different pattern (Figure 1B) and produces peaks up to 12 min. There was a difference in the separation pattern of garlic and black garlic, meaning that during the fermentation process, various changes happened, such as the formation of new compounds that were not initially present in the fresh garlic and were subsequently present in black garlic. This was shown from the peaks at retention times of 1.38, 1.50, 2.09, 2.71, 5.24, and 9.91 min (Figure 1B), and these results are in line with those reported by Kimura et al. (2017) [14]. In addition, several compounds were lost in garlic during the fermentation process. This absence was revealed from the peak retention time in black garlic at 1.06, 1.24, and 8.40 min. Black garlic fermentation also caused an increase in the concentration of various compounds, which can be seen from the high peak intensity at retention times of 1.50, 1.67, 4.33, and 5.24 min (Figure 1), and this follows the report of Qiu et al. (2019) [15].

Each peak on the chromatogram was determined by its mass spectrum. The resulting spectrum in MS1 and MS2 was used to identify compounds, by comparing them with the literature. Three compounds were only detected in fresh garlic, namely L-histidine (1.06 min retention time), alliin (1.24 min retention time), and ajoene (8.40 min retention time). However, 13 compounds were only found in black garlic; and 12 compounds were present in fresh and black garlic extracts (Supplementary Materials, Table S2). Twenty-eight compounds consisted of 13 amino acid groups, seven organosulfur, one phenolic, three organic acid, and four compounds from other functional groups. Analysis of variance showed that all compounds except lysoPC 18:3 and N-acetyl-*S*-allyl-L-cysteine had no significant difference in their levels of compounds in local and imported garlic and black garlic.

### 3.2. Multivariate Data Analysis

**Figure 2:**
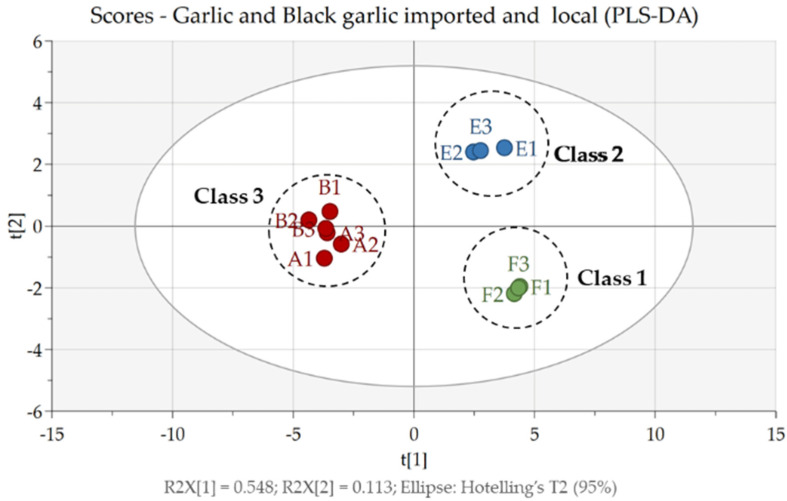
PLS-DA score plot of garlic (Class 1: imported black garlic (F), class 2: local black garlic (E), and class 3: imported and local garlic (A and B)).

In this study, PLS-DA was used as a multivariate data analysis technique with a supervised method that aimed to find the pattern of grouping compounds based on type (garlic and black garlic) and source (local and imported). The PLS-DA score plot (Figure 2) shows that all samples could be classified into three classes, namely class 1 is imported black garlic, class 2 is local black garlic, and class 3 is imported and local garlic. Here, garlic samples by type (black garlic (class 1 and 2) and garlic (class 3)) grouped very well and were in different quadrants, while black garlic samples by location (imported and local) were grouped separately and are in different quadrants. However, the garlic samples were not separated into groups based on their growing location. This showed that the profile of compounds contained in imported and local garlic is the same, while the compound profiles in garlic and black garlic (local and imported) are different. The fermentation of garlic into black garlic caused the compounds contained in the two samples to be different, as previously described in the section on the metabolite profiles of garlic and black garlic extracts. The PLS-DA model with three classes had a good performance, with a cumulative explained variance of R2X = 0.662, R2Y = 0.973, and Q2 = 0.876 [25].

**Figure 3:**
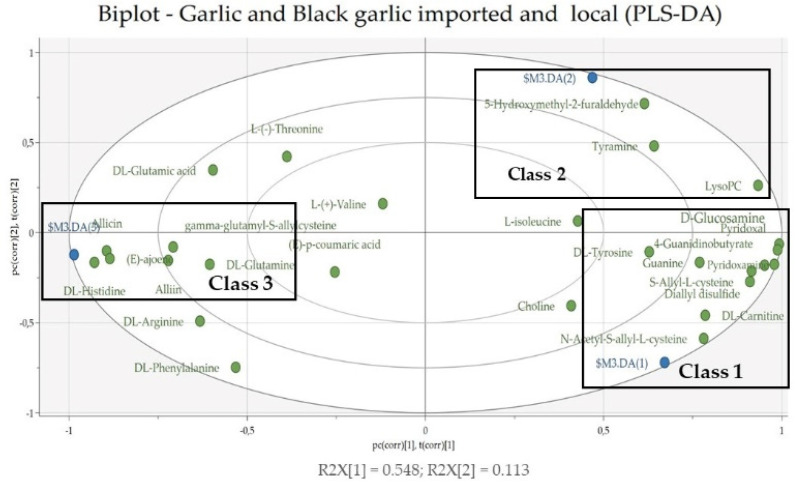
Biplots of imported black garlic (class 1), local black garlic (class 2), and imported and local garlic (class 3).

Validation of the model was done with a random permutation test, 100 times. As seen in Supplementary Materials, Figure S1, the R2Y (green circle) and Q2Y (blue box) values from the permutation analysis (bottom left corner) were lower than the actual R2Y and Q2Y values (top right corner), which indicates that the model has good stability and an absence of overfitting. Validation of the PLS-DA model was followed by a cross-validation test (CV-ANOVA). Based on the cross-validation test (CV-ANOVA), the *p*-value < 0.05 (0.0014066) indicates that the model had good reliability [26]. To determine the compounds that were discriminators in each class, an analysis was carried out using a biplot. Based on the biplot score (Figure 3), the strongest discriminators for class 1 were the compound N-Acetyl-S-allyl-L-cysteine, carnitine, S-Allyl-L-cysteine, diallyl disulfide, pyridoxamine, 4-guanidinobutyric acid, pyridoxal, glucosamine, and guanine. In class 2, the most powerful discriminators were the compounds 5-hydroxymethyl-2-furaldehyde, LysoPC, and tyramine. As for class 3, there were histidine, allicin, ajoene, alliin, γ-Glutamyl-*S*-allyl-cysteine, and DL-glutamine. Discriminator compounds (except compounds from the amino acid group) were then used for a docking analysis, to determine their ability as antigout agents.

### 3.3. Molecular Dynamic Simulation (MD)

**Figure 4:**
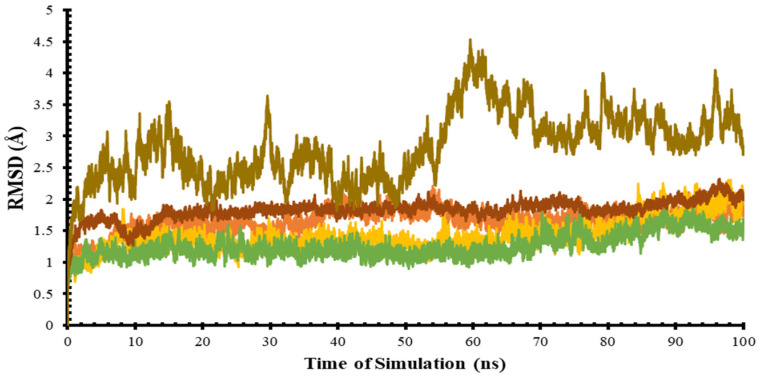
Target protein RMSD values during the 100-ns simulation (Green = ADA, yellow = GDA, orange = XO, brown = NT5C2, and peach = PNP).

In this study, molecular dynamics simulation was used to confirm the protein ensemble, which was then used to calculate the ensemble docking. Analysis of MD results in the root mean square deviation (RMSD), which is the average atomic displacement during a simulation relative to the reference structure, usually the first conformation during the simulation or its crystallographic structure [27]. The RMSD value of the target protein structure is represented on a graph with a simulation time of 100 ns, as shown in Figure 4. Based on Table 1, four target proteins: ADA, GDA, PNP, and XO had RMSD values of approximately ≤2 Å. This shows that the protein structure had a fairly good stability during the simulation and did not deviate from the initial structure, because the difference in the fluctuations in each ns was not too high. Meanwhile, the NT5C2 protein displayed quite large fluctuations for every second but it was considered stable, because the RMSD value was about ≤4 Å.

### 3.4. Ensemble Docking

In this study, we used ensemble docking to determine the ability of compounds in garlic and black garlic to act as antigout treatments. Ensemble docking was chosen because it can describe protein flexibility and in most cases, protein flexibility is under-represented [19], whereas protein flexibility is very important in the computer-aided drug design process, because it can describe the natural state of a protein when it is in the body [19]. We used six target receptors, namely XO, ADA, GDA, PNP, NT5C2-1497, and NT5C2-1498 (3D visualization of the target protein attached in the Supplementary Materials, Figure S2). The molecular dynamics simulation produced ten protein conformations, representing a conformation every 10 ns during the simulation (Supplementary Materials, Figure S3). Then, the ten conformations of the target receptor were docked with marker compounds based on the results of the multivariate analysis. The results of the ensemble docking are revealed in Table 2.

Based on the results of the ensemble docking analysis, the sequence of compounds with the best binding affinity for the target receptor was pyridoxamine, which is a non-identifying organic compound found abundantly in black garlic. These compounds can inhibit all target receptors that play a role in the process of uric acid biosynthesis. D-Glucosamine ranked second after pyridoxamine. D-Glucosamine is a non-identifying organic compound that is also found in abundance in black garlic. Based on the results of the ensemble docking analysis, D-glucosamine can be developed as an antigout candidate because it can inhibit four target receptors, namely XO, ADA, GDA, and NT5C2-1498. This was evidenced by the binding energy, which was very negative compared to the original substrate and commercial compounds. Ajoene, which is a discriminator for garlic, has potential as a natural inhibitor for inhibiting XO, ADA, and NT5C2-1497 enzymes. This was evidenced by the binding energy value, which was more negative than the original substrate and also commercial compounds, namely allopurinol (XO), fludarabine (NT5C2), and EHNA (ADA) [28,29,30]. Pyridoxal was the only compound that only inhibited two target receptors, namely XO and ADA, with binding affinity values of −5.01 ± 0.53 kcal/mol and −5.12 ± 0.51 more negative than commercial substrates and compounds. Alliin and N-acetyl-S-allyl-L-cysteine can be developed as antigout candidates, because they can inhibit different target receptors, namely ADA (alliin) and NT5C2-1497 (N-acetyl-S-allyl-L-cysteine and 4-guanidinobutyric acid). This was evidenced by the very negative binding energy value compared to the original substrate and commercial compounds from both receptors, with binding affinity values of −4.72 ± 0.47 kcal/mol (ADA-alliin complex) and −4.36 ± 0.28 kcal/mol (NT5C2-1497-N-acetyl-S-allyl-L-cysteine complex).

**Figure 5:**
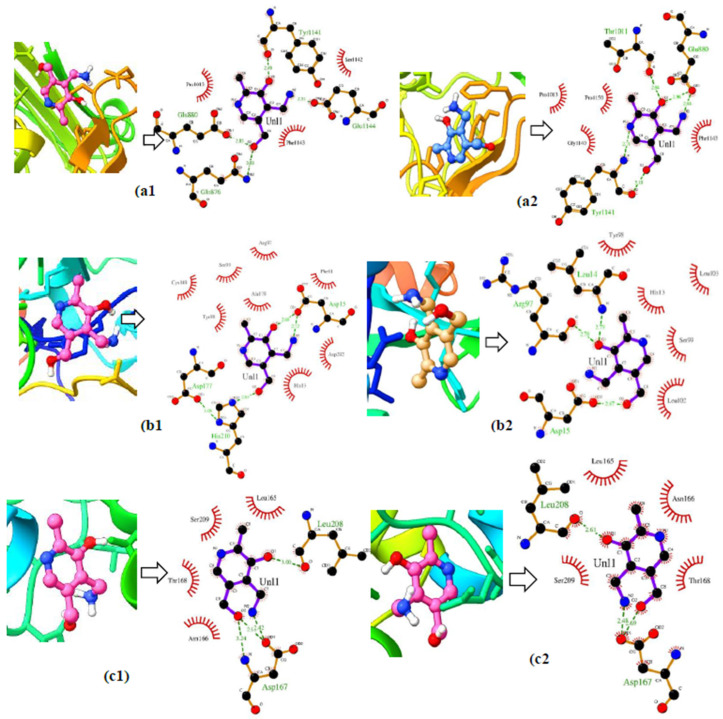
3D and 2D visualization of the early conformational (**a1**,**b1**,**c1**) and late conformational ligand–receptor complexes (**a2**,**b2**,**c2**).

The visualization of the 2D and 3D interactions of the ligand–receptor complex from the ensemble docking illustrates the differences in the number and type of amino acid residues that interacted in the initial conformation (0) and final conformation (Figure 5). The XO–pyridoxamine complex in the initial conformation (0) (Figure 5a1) has three hydrophobic interactions at the amino acid residues Pro1013, Phe1143, and Ser1142, and four hydrogen bond interactions at residues Tyr1141, Glu1144, Gln876, and Glu880. While in the final conformation (Figure 5a2), the hydrophobic interactions increased by four, which occurred at the amino acid residues Pro1013, Phe1143, Ser1140, Pro1150; and the hydrogen-bonding interactions increased by five, namely at residues Thr1011, Glu880 (a), Glu880 (b), Tyr1141 (a), and Tyr1141(b). The same thing happened to the other protein–ligand complexes, namely the ADA–pyridoxamine (Figure 5b1,b2) and GDA–pyridoxamine complexes (Figure 5c1,c2).

The difference in the number of interacting amino acid residues affected the binding energy (BE) value (Supplementary Materials, Table S3). The difference in the number and type of interacting amino acid residues was caused by the occurrence of protein flexibility every second, causing the test ligands to bind to different amino acid residues at different distances. This proves that the ensemble docking analysis could consider the flexibility of the protein during the simulation.

### 3.5. Screening of Bioactive Compounds as Drug Candidates

Compounds that have potential as antigout treatments, based on the results of the ensemble docking, were tested for compliance with Lipinski rules and ADMET properties. Lipinski’s rule showed the permeability and ability of the test ligand was well absorbed orally. Lipinski test parameters included molecular weight (MW) < 500 Da, octanol/water partition coefficient (A log *p*) < 5, number of hydrogen bond donors (HBD) < 5, and number of hydrogen bond acceptors (HBA) < 10 [31]. Lipinski’s rule has a tolerance limit that is allowed to violate one rule [32]. At the same time, an ADMET test was used to determine the absorption, distribution, metabolism, excretion, and toxicity. This indicator has become important in research related to drug development [33]. All test ligands and commercial ligands obeyed the Lipinski rule, and all test ligand compounds had the potential to be developed into drug candidates, because they have good bioavailability and high percentage of them could be absorbed. In addition, the intestine and digestive system do not have the potential to cause cancer and did not cause mutations in the test bacteria (Table 3). Positive and negative signs in the following table indicate whether or not certain parameters occurred.

## 4. Discussion

The use of natural products is a promising alternative in treating various diseases, one of which is gout. Besides being easy to obtain, natural products are also believed to be safer and have provided significant results in lowering uric acid levels in the blood, as reported by Daoudi et al. (2020) [34]. They have become the focus of research on utilizing other natural products, namely garlic bulbs and black garlic. Garlic (*Allium sativum*) is one of the oldest herbs and has been used for thousands of years ago in traditional medicine. However, science and technology have advanced over time, and a unique processed garlic product has emerged, namely black garlic. Black garlic is a processed product of raw garlic or shallots heated at high temperatures [35]. Based on the results of our study, during the fermentation process, several events occurred, such as the increase in the levels of several compounds found in black garlic, the loss of compounds found in garlic, and the emergence of new compounds that were not previously present in garlic; and this is in line with the reports of Choi et al. (2014) and Qiu et al. (2019) [15,36]. This can also be seen from the results of our multivariate analysis using PLS-DA. In which three class groups were formed, with classes 1 and 2 being black garlic and class 3 being garlic; and the three classes separated very well. This shows that the two types of garlic have different compound profiles. As far as we know, no one has reported a multivariate analysis of garlic and black garlic samples; therefore, our results can serve as a reference and information for other researchers.

Uric acid in the body is produced through the action of several enzymes, such as xanthine oxidase (XO) [5], adenine deaminase (ADA), guanine deaminase (GDA) [7], purine nucleoside phosphorylase (PNP) [8], and 5-nucleotidase II. (NT5C2) [9]. The mechanism of uric acid formation can be seen in the Supplementary Materials, Figure S4. 5-Nucleotidase II is an enzyme that catalyzes the dephosphorylation of ribonucleoside monophosphate into inorganic nucleosides and phosphates. The resulting nucleosides are guanosine, xanthosine, inosine, and adenosine. The four nucleosides are substrates for the purine nucleoside phosphorylase and adenine deaminase, in producing the following products: Purine nucleoside phosphorylase is an enzyme that plays a role in purine catabolism pathways. This enzyme catalyzes the reversible phosphorylation of N-ribosidic nucleoside bonds and produces purine bases and ribose 1-phosphate. The purine bases formed are guanine, xanthine, and hypoxanthine, substrates for guanine deaminase and xanthine oxidase. Furthermore, the guanine deaminase enzyme converts guanine into xanthine, and the xanthine oxidase enzyme converts hypoxanthine to xanthine and becomes uric acid (Supplementary Materials, Figure S4).

The process of uric acid biosynthesis is not solely carried out by one or two enzymes, many enzymes play a role during the biosynthetic process. As shown in Figure S4, five enzymes work continuously to produce the final product, in the form of uric acid. A single-drug target approach to treat gout may be less effective, so targeting multiple receptors involved in uric acid biosynthesis is an ideal strategy in this case. The previously mentioned five receptors were used as target receptors. The hope is that inhibiting all enzymes that play a role in the biosynthesis of uric acid can significantly reduce the level of uric acid. To our knowledge, there have been no previous reports of treating gout through a multi-targeted approach. Even the drugs commonly used in gout patients to treat gout, allopurinol and febuxostat [10], only work on one target receptor: xanthine oxidase. Thus, in this study, we tried to dock the bioactive components of garlic to XO, ADA, GDA, PNP, and NT5C2 receptors, all of which play a role in uric acid biosynthesis, using an ensemble docking approach. Ensemble docking was chosen, because it can describe the protein’s natural state in the body when experiencing flexibility [37]. As we reported, based on the visualization results of the interaction of the ligand–receptor complex in the initial conformation with the final conformation, there were differences in the number, spacing, and types of interacting amino acid residues (Supplementary Materials, Table S3). This suggests that our ensemble docking approach can take protein flexibility into account.

Based on our results, several compounds can only be found in garlic, while others are only found in black garlic, with concentrations that are not significantly different between the local and the imported samples (Supplementary Materials, Table S2). These compounds can block almost all target receptors. The compounds found only in garlic are alliin and ajoene, while N-acetyl-*S*-allyl-L-cysteine, pyridoxal, pyridoxamine, 4-guanidinobutyric acid, and D-glucosamine were only found in black garlic. Pyridoxamine is the only compound found to be a bioactive molecule that interacts with all target proteins. Based on our study, the compounds we found in garlic that have potential as antigout agents were different from those reported by Johnson et al. (2018) and Setiawan et al. (2018) [18,38]. The docking approach used was also different; we used ensemble docking to determine the potential compounds, while their report used a simple docking approach. Physicochemical properties and pharmacokinetic profiles (ADMET) indicated that the previously mentioned bioactive molecules could be developed as drug candidates, because they scored well in each test parameter. All test compounds can be recommended for clinically testing, for the inhibition of the six target receptors. Our results can be used as a reference for standardizing the quality of black garlic, in developing multi-target antigout herbal medicines.

## 5. Conclusions

Using an ensemble docking approach, we gained structural insights into the possible binding modes of bioactive molecules from garlic and black garlic to the target receptors that play a role in uric acid biosynthesis. Pyridoxamine binds best to all target receptors; this compound is abundant in black garlic. It is followed by D-glucosamine, which was well tethered to all receptors, except PNP; and NT5C2-1497 is also found abundantly in black garlic. Ajoene blocks three target receptors, namely XO, ADA, and NT5C2-1497. Ajoene is abundant in garlic. Pyridoxal can inhibit XO and ADA and is detected abundantly in black garlic. Alliin, N-acetyl-*S*-allyl-L-cysteine, and 4-guanidinobutyric acid can only block one receptor, with alliin found in abundance in garlic, and the following two compounds are found in abundance in black garlic. Black garlic would be better as a multi-target antigout drug, because of its abundant bioactive molecules. Multivariate analysis succeeded in classifying the samples into three classes: class 1 imported black garlic, class 2 local black garlic, and class 3 imported and local garlic, which reflected the similarities and differences between the discriminator compounds.

## Figures and Tables

**Table 1:** RMSD value of the target protein.

Protein	Minimum and Maximum (Å)	Difference in Distance (Å)
Xanthine oxidase (XO)	0.48–2.30	1.82
Adenine deaminase (ADA)	0.45–2.00	1.55
Guanine deaminase (GDA)	0.48–2.35	1.87
Purine nucleoside phosphorylase (PNP)	0.48–2.25	1.77
5-Nucleotidase II (NT5C2)	0.70–4.50	3.80

**Table 2:** Binding energy of 13 compounds, resulting from ensemble docking using 10 conformations.

Name	Binding Energy (kcal/mol)					
	XO	ADA	GDA	PNP	NT5C2-1497	NT5C2-1498
Diallyl thiosulfinate (Allicin)	−4.74 ± 0.52	−4.43 ± 0.38	−4.46 ± 0.15	−4.28 ± 0.23	−3.94 ± 0.24	−3.66 ± 0.18
Alliin	−4.15 ± 0.38	−4.72 ± 0.47	−4.43 ± 0.24	−4.81 ± 0.26	−3.61 ± 0.36	−3.60 ± 0.47
*S*-allyl-L-cysteine	−4.04 ± 0.39	−4.51 ± 0.29	−4.12 ± 0.16	−4.54 ± 0.20	−3.72 ± 0.20	−3.54 ± 0.27
Diallyl disulfide	−2.80 ± 0.11	−3.19 ± 0.33	−3.39 ± 0.13	−2.94 ± 0.22	−3.27 ± 0.20	−3.05 ± 0.06
γ-Glutamyl-*S*-allyl-cysteine	−2.99 ± 0.42	−4.10 ± 0. 48	−2.94 ± 0.62	−2.30 ± 0.57	−3.47 ± 0.27	−3.76 ± 0.72
N-acetyl-*S*-allyl-L-cysteine	−3.85 ± 0.23	−3.56 ± 0. 34	−3.80 ± 0.41	−4.36 ± 0.33	−4.36 ± 0.28	−4.25 ± 0.31
(*E*)-Ajoene	−4.91 ± 0.12	−5.32 ± 0. 24	−4.94 ± 0.22	−5.16 ± 0.28	−4.58 ± 0.26	−4.48 ± 0.28
Pyridoxal	−5.01 ± 0.53	−5.12 ± 0. 51	−4.90 ± 0.27	−4.71 ± 0.15	−4.36 ± 0.26	−4.64 ± 0.16
Pyridoxamine	−5.26 ± 0.24	−6.78 ± 0. 29	−5.53 ± 0.29	−6.06 ± 0.15	−4.66 ± 0.30	−4.68 ± 0.41
DL-Carnitine	−2.39 ± 0.23	−3.17 ± 0.30	−2.52 ± 0.33	−2.65 ± 0.30	−3.44 ± 0.21	−3.48 ± 0.44
4-Guanidinobutyric acid	−4.65 ± 0.43	−3.60 ± 0.52	−3.66 ± 0.21	−4.58 ± 0.36	−4.79 ± 0.47	−4.17 ± 0.49
D-Glucosamine	−5.23 ± 0.34	−6.03 ± 0.39	−5.27 ± 0.27	−5.83 ± 0.23	−4.03 ± 0.53	−4.67 ± 0.35
5-hydroxymethyl-2-furaldehyde	−3.56 ± 0.29	−3.70 ± 0.26	−3.83 ± 0.20	−3.92 ± 0.19	−3.72 ± 0.23	−3.34 ± 0.21
Allopurinol (commercial) Xanthine (substrate) Hipoxanthine (substrate)	−4.82 ± 0.25					
	−4.92 ± 0.36					
	−4.72 ± 0.38					
Erythro-9-(2-hydroxy-3-nonyl)adenine [EHNA] (commercial) Adenosine (substrate)		−4.73 ± 0.42				
		−4.52 ± 0.58				
Azepinomycin (commercial) Guanine (substrate)			−4.86 ± 0.28			
			−5.27 ± 0.16			
Ulodesine (commercial)				−6.03 ± 0.68		
Guanosine (substrate)				−5.78 ± 0.67		
Inosine (substrate)				−4.84 ± 0.40		
Xanthosine (substrate)				−5.63 ± 0.55		
Fludarabine (commercial) Adenosine monophosphate (substrate) Guanosine monophosphate (substrate) Inosine monophosphate (substrate) Xanthosine monophosphate (substrate)					−4.20 ± 0.52	−4.04 ± 0.49
					−4.18 ± 0.37	−4.67 ± 0.33
					−3.44 ± 0.36	−4.67 ± 1,13
					−4.73 ± 0.47	−4.73 ± 0.80
					−4.66 ± 0.51	−4.63 ± 0.45

**Table 3:** Physicochemical Parameters and ADMET Test and Commercial Ligands.

Ligand	Physicochemical Parameters				ADMET Parameters				
	MW ^a^ (<500 g mol^−1^)	LogP ^b^ (<5)	HBD ^c^ (<5)	HBA ^d^ (<10)	Bioavaibility (Score)	Human Intestinal Absorption (HIA)	AMES Mutagenesis	Carciogenicity	LD_50_ (mg/kg) (Predicted Toxicity Class)
Allopurinol	136.11	−0.19	2	4	GB (0.55)	HIA (+)	AMES (−)	NC	1000 (IV)
Ulodesine	264.28	−0.80	4	6	GB (0.55)	HIA (+)	AMES (−)	NC	1000 (IV)
Erythro-9-(2-Hydroxy-3-nonyl)adenin [EHNA]	277.37	2.30	3	5	GB (0.55)	HIA (+)	AMES (−)	NC	450 (IV)
Azepinomycin	168.16	−1.12	4	5	GB (0.55)	HIA (+)	AMES (−)	NC	2032 (V)
Fludarabine	285.24	−2.84	4	9	GB (0.55)	HIA (+)	AMES (−)	NC	13 (II)
Alliin	177.22	−0.67	2	3	GB (0.55)	HIA (+)	AMES (−)	NC	8000 (VI)
N-Acetyl-S-Allyl-L-Cysteine	203.26	0.49	2	3	GB (0.56)	HIA (+)	AMES (−)	NC	4000 (V)
(E)-Ajoene	234.41	3.00	0	3	GB (0.55)	HIA (+)	AMES (−)	NC	1600 (IV)
Pyridoxal	167.16	0.40	2	13	GB (0.55)	HIA (+)	AMES (−)	NC	1120 (IV)
Pyridoxamine	168.20	0.05	3	4	GB (0.55)	HIA (+)	AMES (−)	NC	5100 (VI)
4-Guanidinobutyric acid	145.16	−0.88	3	2	GB (0.55)	HIA (+)	AMES (−)	NC	12,680 (VI)
D-Glucosamine	179.17	−2.55	5	19	GB (0.55)	HIA (+)	AMES (−)	NC	1000 (IV)

Information: ^a^ = molecular weight, ^b^ = octanol water partition coefficient, ^c^ = hydrogen bond donor, ^d^ = hydrogen acceptor donor, GB = good bioavailability, NGB = not good bioavailability, NC = noncarcinogenicity.

## Data Availability

Data are contained within the article and Supplementary Material.

## Supplementary Materials

The following supporting information can be downloaded at: https://www.mdpi.com/article/10.3390/life12081131/s1, Figure S1: Permutation test for the PLS-DA model with (a) imported black garlic; (b) local black garlic; (c) imported and local garlic; Figure S2: Target protein 3D picture; Figure S3: Protein conformation from ensemble docking; Figure S4: Uric acid biosynthesis mechanism; Table S1: Test and reference ligands; Table S2: Putative identification of metabolites present in extracts of fresh garlic (FG) (import and local) and black garlic (BG) (import and local) using LC-MS/MS in positive ionization mode; Table S3: ligand–receptor complex interactions resulting from ensemble docking.
