# Supplementary material for: Bioactive Compounds in Garlic (Allium sativum) and Black Garlic as Antigout Agents, Using Computer Simulation
Source: Life (Basel). 2022 Jul 27;12(8):1131. doi: 10.3390/life12081131 (PMC9409881; doi:10.3390/life12081131)
Supplement: Supplementary file 1 [file life-12-01131-s001.zip › life-1802270-supplementary.pdf]

Bioactive Compounds in Garlic (*Allium sativum*) and Black Garlic as Antigout Based on in Silico Approach

Supplementary Material

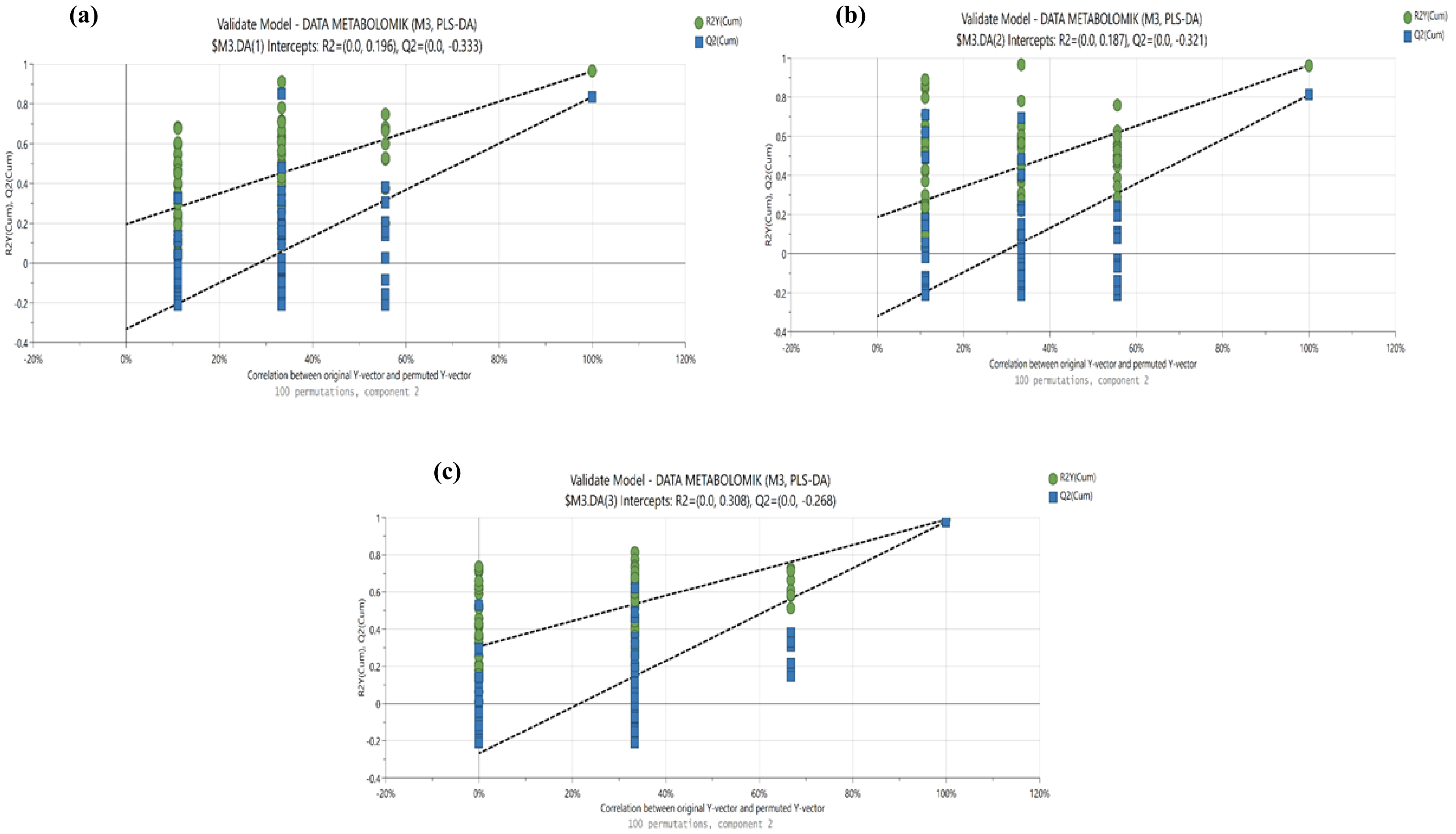

Figure S1 Permutation test for the PLS-DA model with (a) imported black garlic; (b) local black garlic; (c) imported and local garlic

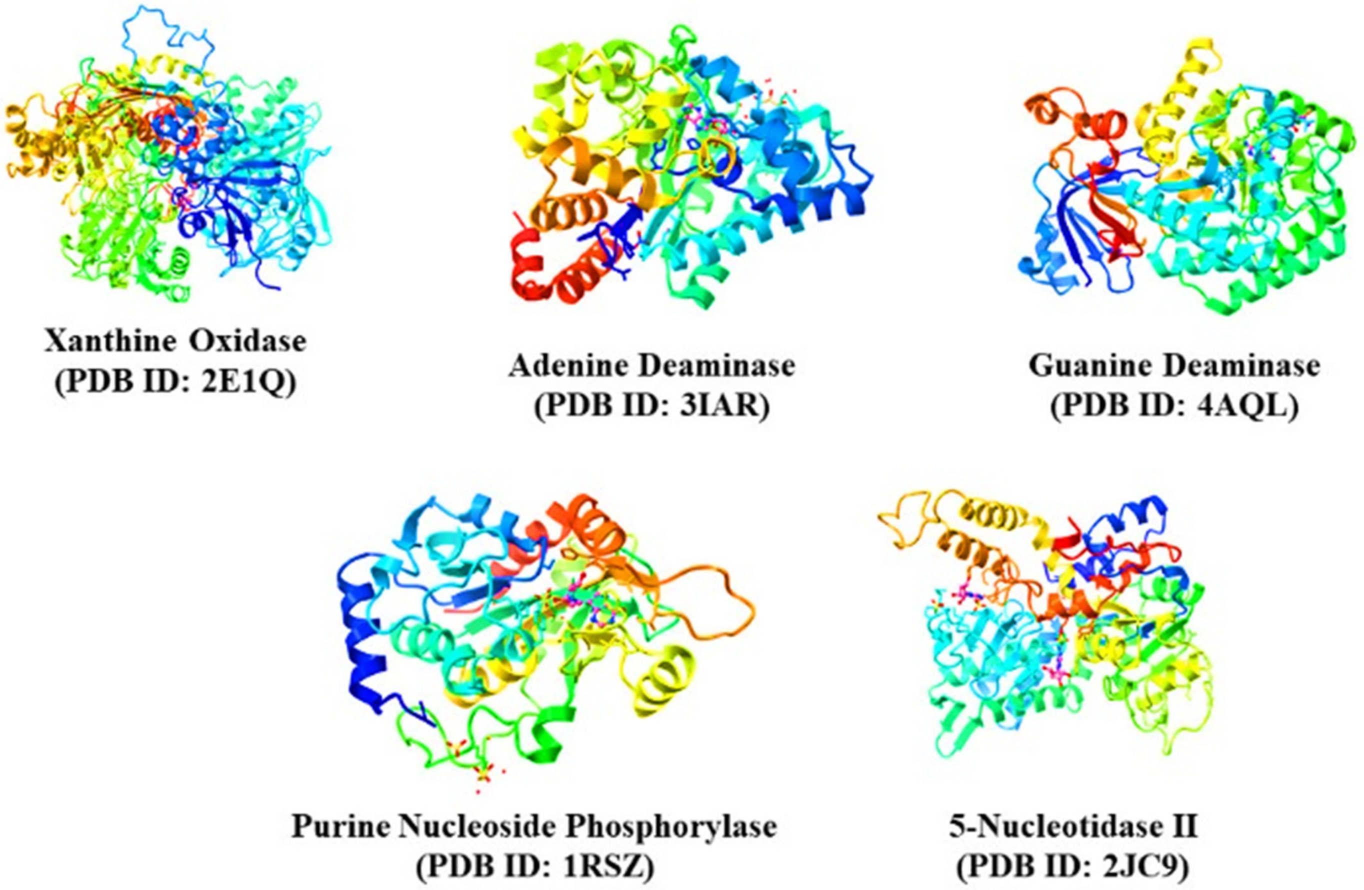

Figure S2 Target protein 3D picture

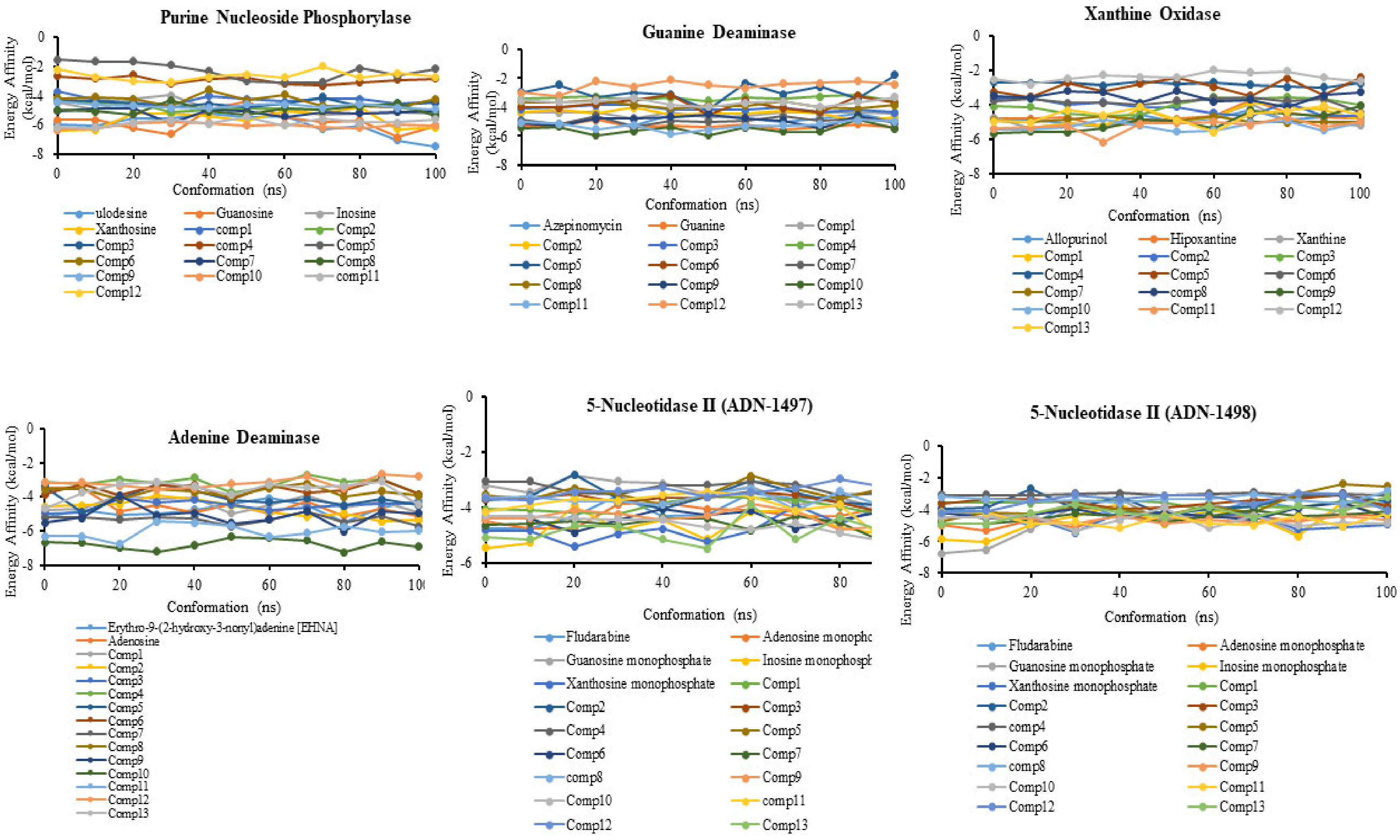

Figure S3 Protein conformation from ensemble docking

Information

Ligand	Name
Comp1	Diallyl thiosulfinate (Allicin)
Comp2	Alliin
Comp3	***S***-Allyl-L-cysteine
Comp4	Diallyl disulfide
Comp5	***γ***-Glutamyl-***S***-Allyl-Cysteine
Comp6	N-Acetyl-***S***-Allyl-L-Cysteine
Comp7	(*E*)-Ajoene
Comp8	5-hydroxymethyl-2-furaldehyde
Comp9	Pyridoxal
Comp10	Pyridoxamine
Comp11	D-Glucosamine
Comp12	DL-Carnitin
Comp13	4-Guanidinobutyric acid

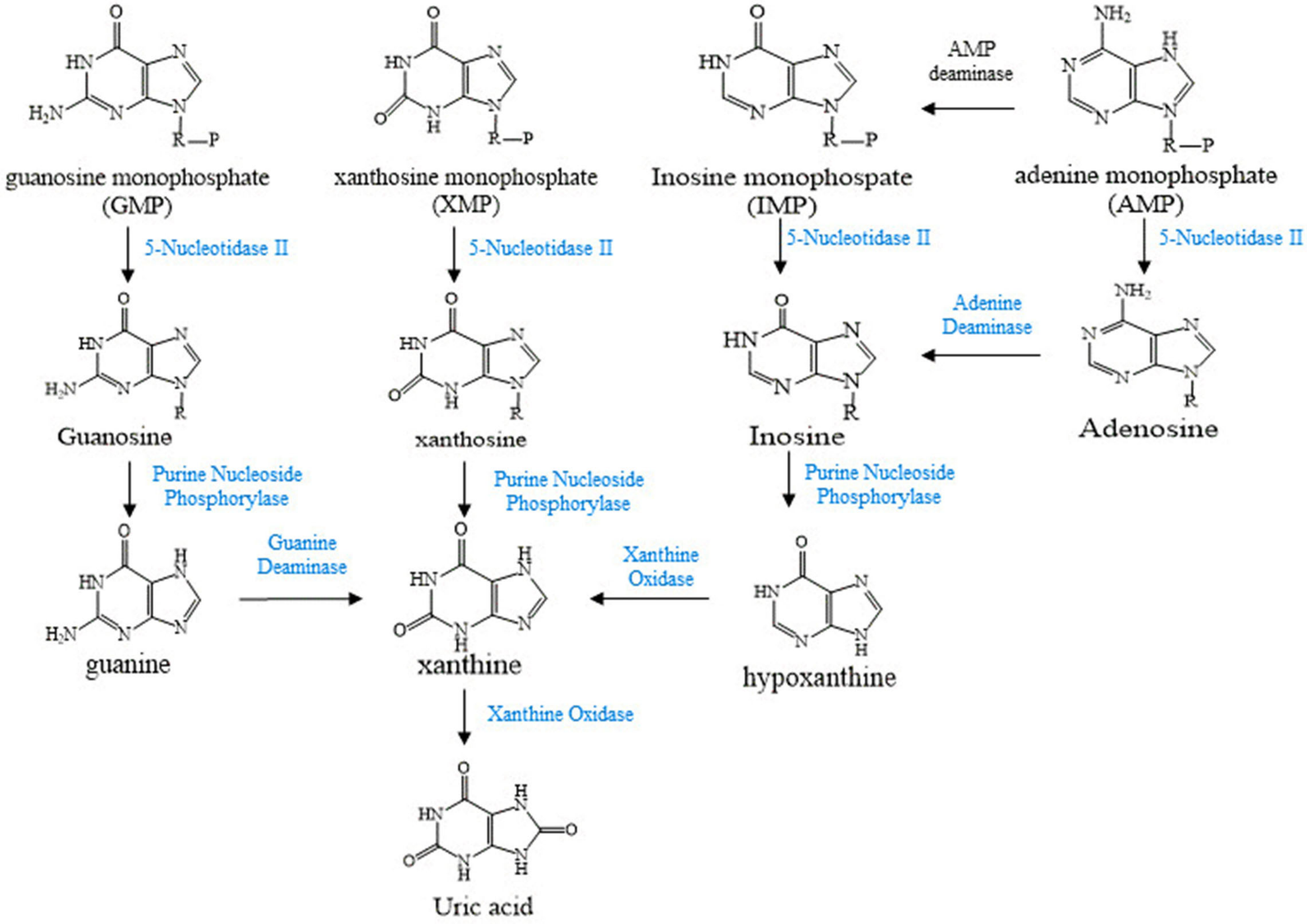

Figure S4 Uric acid biosynthesis mechanism

Table S1 Test and reference ligands

Ligands	Reference ligands	
	Substrate	Commercil
1. Diallyl thiosulfinate (Allicin)	Xanthine	Allopurinol
2. Alliin	Hypoxanthine	Erythro-9-(2-hydroxy-nonyl)adenine [EHNA]
3. *S*-allyl-L-cysteine	Adenosine	Azepinomycin
4. Diallyl disulfide	Guanine	Ulodesine
5. **γ**-Glutamyl-*S*-allyl-cysteine	Guanosine	Fludarabine
6. N-acetyl-*S*-allyl-L-cysteine	Inosine	
7. (*E*)-Ajoene	Xanthosine	
8. 5-hydroxymethyl-2-furaldehyde	Adenosine monophosphate	
9. Pyridoxal	Guanosine monophosphate	
10. Pyridoxamine	Inosine monophosphate	
11. DL-Carnitine	Xanthosine monophosphate	
12. 4-Guanidinobutyric acid		
13. D-Glucosamine		

Table S2 Putative identification of metabolite present in extract fresh garlic (FG) (import and local) and black garlic (BG) (import and local) using LC-MS/MS in positive ionization mode

RT [min]	Metabolite name	Molecul formula	MW	Error mass [ppm]	MS-MS2 Fragmentation	Abudance (%)				Sample			
						FG Import	BG Import	FG Local	BG Local	FG Import	BG Import	FG Local	BG Local
						**Amino Acid**							
1.06	L-histidine	$C_6H_9N_3O_2$	155.0691	-2,71	156.07616, 110.07117, 83.06061	0,8082 ± 0,20073 [b]	0,0398 ± 0,1579 [a]	0,7234 ± 0,2055 [b]	0,0470 ± 0,1553 [a]	√	*	√	*
1.29	Valine	$C_5H_{11}NO_2$	117,0788	-1,43	118.08585, 72.08111	1,6213 ± 2,75194 [a]	1,0980 ± 1,71216 [a]	1,7147 ± 1,46967 [a]	3,3720 ± 2,85011 [a]	√	√	√	√
2.09	D-(+)-Proline	$C_5H_9NO_2$	115,0632	-1,15	116.07034, 70.06540	0,0240 ± 0,1229 [a]	0,2147 ± 0,17182 [b]	0,0140 ± 0,1217 [a]	0,3673 ± 0,10471 [b]	*	√	*	√
4.33	L-Phenylalanine	$C_9H_{11}NO_2$	165,0783	-3,88	166.08568, 120.08053	0,6240 ± 0,1778 [b,c]	0,6847 ± 0,10281 [c]	0,4967 ± 0,10372 [a,b]	0,4697 ± 0,03317 [a]	√	√	√	√
1.08	L-(+)-Arginine	$C_6H_{14}N_4O_2$	174,1111	-3,54	175.11852, 158.09204, 130.09726	24,3823 ± 3,0774 [a,b]	30,3920 ± 4,6530 [b,c]	34,8547 ± 4,75181 [c]	17,9117 ± 1,42333 [a]	√	*	√	*
1.51	L-Isoleucine	$C_6H_{13}NO_2$	131,0943	-2,77	132.10127, 86.09663	2,9360 ± 3,19383 [a]	6,4793 ± 3,73368 [a]	1,4097 ± 2,41048 [a]	8,2017 ± 9,89227 [a]	√	√	*	√
1.50	Guanine	$C_5H_5N_5O$	151,049	-2,83	152.05818, 135.02985	0,0583 ± 0,0089 [a,b]	0,3004 ± 0,03957 [c]	0,0155 ± 0,01541 [a]	0,2334 ± 0,1989 [b,c]	*	√	*	√
1.14	DL-Glutamine	$C_5H_{10}N_2O_3$	146,0688	-2,58	147.07600, 130.04962, 84.04459	0,7520 ± 0,51125 [a]	0,7120 ± 0,17724 [a]	1,0733 ± 0,48564 [a]	0,7707 ± 0,4015 [a]	√	√	√	√
1.13	L-Glutamic acid	$C_5H_9NO_4$	147,0528	-2,78	148.0615, 130.04961, 102.05501, 84.04458	0,6780 ± 0,24318 [a]	0,677 2 ± 0,18988 [a]	1, 4570 ± 0,14490 [b]	1,6005 ± 0,23511 [b]	*	*	√	√
1.12	L-(-)-Threonine	$C_4H_9NO_3$	119,0582	-0,47	120.06552, 102.05497	0,1976 ± 0,03290 [a]	0,2101 ± 0,0232 [a,b]	0,3791 ± 0,17768 [b,c]	0,4906 ± 0,02960 [c]	√	√	√	√
2.08	L-Tirosine	$C_9H_{11}NO_3$	181,0732	-3,68	182.08064, 136.07593, 119.04916	0,0070 ± 0,0010 [a]	0,2940 ± 0,31090 [a]	0,0053 ± 0,00289 [a]	0,2423 ± 0,14468 [a]	*	√	*	√

RT [min]	Metabolite name	Molecul formula	MW	Error mass [ppm]	MS-MS2 Fragmentation	Abudance (%)				Sample			
						FG Import	BG Import	FG Local	BG Local	FG Import	BG Import	FG Local	BG Local
2.51	Tyramine	$C_8H_{11}NO$	137,0838	-2,11	138.09076, 94.06528, 65.03899	0,0017 ± 0,00115 [a]	0,0540 ± 0,00781 [a]	0,0020 ± 0,00173 [a]	0,1733 ± 0,07295 [b]	*	√	*	√
9.91	LysoPC (18:3(9Z,12Z,15Z))	$C_{26}H_{48}NO_7P$	517,3141	-5,38	518.32190, 414.03827	0,0030 ± 0,00173 [a]	0,1563 ± 0,04868 [b]	0,0023 ± 0,00153 [a]	0,3023 ± 0,12231 [c]	*	√	*	√√
						**Organic sulfur**							
6.66	Diallyl thiosulfinate (Allicin)	$C_6H_{10}OS_2$	162,0167	-3,98	163.02380, 73.01093	20,3953 ± 8,15248 [b]	0,0073 ± 0,00231 [a]	18,8753 ± 5,26397 [b]	0,1017 ± 0,08297 [a]	√	*	√	√
1.24	Alliin	$C_6H_{11}NO_3S$	177,0452	-4,49	178.05237, 159.07625, 117.07405	16,4463 ± 8,45083 [b]	1,4203 ± 0,85162 [a]	9,2650 ± 7,96354 [a,b]	2,0773 ± 1,26253 [a]	√	*	√	*
1.67	***S***-Allyl-L-cysteine	$C_6H_{11}NO_2S$	161,0504	-3,75	162.05794, 145.03139, 73.01095	0,8373 ± 0,06824 [a]	19,2553 ± 6,92564 [b]	0,4540 ± 0,26678 [a]	14,3533 ± 0,30994 [b]	√	√	√	√
5.24	Diallyl disulfide	$C_6H_{10}S_2$	146,0223	-0,83	147.02942, 104.98278, 73.01100	0,0003 ± 0,00058 [a]	0,0487 ± 0,01823 [b]	0,0010 ± 0,00173 [a]	0,0400 ± 0,01311 [b]	*	√	*	√
3.99	***γ***-Glutamyl-***S***-Allyl-Cysteine	$C_{11}H_{18}N_2OS$	290,0939	0,76	291.09979, 145.03149, 73.02099	13,2880 ± 9,59068 [a]	2,7293 ± 1,55445 [a]	12,5223 ± 9,59599 [a]	4,6327 ± 3,95886 [a]	√	√	√	√
2.79	N-Acetyl-***S***-Allyl-L-Cysteine	$C_8H_{13}NO_3S$	203,061	-2,95	204.06837, 186.05811, 112.07571	0,0002 ± 0,00023 [a]	0,2442 ± 0,03901 [c]	0,0001 ± 0,0006 [a]	0,0669 ± 0,02199 [b]	*	√√	*	√
8.40	(*E*)-Ajoene	$C_9H_{14}OS_3$	234,0199	-3,5	235.02629, 195.04268	0,3617 ± 0,11064 [b]	0,0010 ± 0,00100 [a]	0,3140 ± 0,18824 [b]	0,0037 ± 0,00306 [a]	√	*	√	*
RT [min]	Metabolite name	Molecul formula	MW		MS-MS2 Fragmentation	Abudance (%)				Sample			

				Error mass [ppm]		FG Import	BG Import	FG Local	BG Local	FG Import	BG Import	FG Local	BG Local
						**Phenolic**							
1,52	*p*-Coumaric acid	$C_9H_8O_3$	164,0468	-3,21	165.05394, 147.04356, 120.08871	2,7060 ± 2,87608 ^{a}	1,4903 ± 1,65644 ^{a}	1,0807 ± 0,37321 ^{a}	1,4493 ± 1,19021 ^{a}	√	√	√	√
						**Organic acid**							
2.31	Pyridoxal	$C_8H_9NO_3$	167,0577	-3,02	168.06493, 122,05997, 94.06530	0,0020 ± 0,0010 ^{a}	0,2933 ± 0,04576 ^{b}	0,0017 ± 0,00115 ^{a}	0,3270 ± 0,10058 ^{b}	*	√	*	√
2.87	Pyridoxamine	$C_8H_{12}N_2O_2$	168,0894	-3,14	169.09628, 151.08621, 121.08408	0,0010 ± 0,00035 ^{a}	0,1317 ± 0,01801 ^{b}	0,0007 ± 0,00067 ^{a}	0,1247 ± 0,02775 ^{b}	*	√	*	√
2.95	5-Hydroxymethyl-2-furadehide	$C_6H_6O_3$	126,0315	-1,71	127.03864, 109.02843, 81.03377	0,0200 ± 0,00781 ^{a}	0,2550 ± 0,26831 ^{a}	0,0233 ± 0,01401 ^{a}	1,9093 ± 0,53938 ^{b}	*	√	*	√
						**Other**							
2.20	Choline	$C_5H_{13}NO$	103,0977	0,07	104.10687, 60.08119	13,5257 ± 2,83329 ^{a}	22,9997 ± 2,81381 ^{b,c}	15,0937 ± 2,98501 ^{a,b}	30,0190 ± 7,49419 ^{c}	√	√	√	√
1.40	DL-Carnitin	$C_7H_{15}NO_3$	161,1047	-2,99	162.11185, 146.09215, 104.07071, 60.05610	0,1563 ± 0,06741 ^{a}	0,4263 ± 0,00451 ^{b}	0,0733 ± 0,02836 ^{a}	0,3910 ± 0,16029 ^{b}	*	√	*	√
1.38	4-Guanidinobutyric acid	$C_5H_{11}N_3O_2$	145,0846	-3,47	146.09178, 87.04431, 60.05610	0,1147 ± 0,05107 ^{a}	3,5023 ± 0,85523 ^{b}	0,0997 ± 0,02829 ^{a}	3,5920 ± 1,62896 ^{b}	*	√	*	√
2.71	D-Glucosamine	$C_6H_{13}NO_5$	179,0787	-3,77	180.08491, 162.07579, 84.04467	0,0510 ± 0,01082 ^{a}	5,8823 ± 0,66678 ^{b}	0,0473 ± 0,04908 ^{a}	6,7293 ± 1,68879 ^{b}	*	√	*	√

Numbers followed by the same letter represent values that are not significantly different ($p > 0.05$), √ (Detected), * (Not Detected)

Table S3 ligand-receptor complex interactions result ensemble docking

Compunds (Conformation)	BE (kcal/mol)	Hydrophobic interactions	Hydrogen bond interactions (distance, Å)
		**XDH**	
(*E*)-Ajoene (0)	-4,92	Gln872, Asp873, Leu874, Val1012, Phe1010, Leu1015, Ala911, Ile878, Phe915, Ser875, Gln876	Ser877 (2.68)
(*E*)-Ajoene (8)	-5,03	Asn769, Thr1075, Lys772, Ala1079, Val803, Arg881, Phe1010, Ser877, Pro1077, Leu874, Phe915, Ala1080, Leu1015, Met771	-
Pyridoxamine (0)	-5,42	Pro1013, Phe1143, Ser1142	Tyr1141 (2.49), Glu1144 (2.51), Gln876 (3.03), Glu880 (2.85)
Pyridoxamine (5)	-5,56	Pro1013, Phe1143, Ser1140, Pro1150	Thr1011 (2.84), Glu880 (2.96), Glu880 (2.44), Tyr1141 (3.10), Tyr1141 (2.71)
Pyridoxal (0)	-5,67	Ala1259, Val1260, Gly1261, Met1039, Gln1041, Phe799, Thr1078	Ala1084 (3.11), Ser1081 (2.59), Glu1262 (2.59), Ala1079 (2.87), Ser1083 (3.26)
D-Glucosamine (0)	-5,39	Pro1013, Glu1144, Ser1142, Val1012	Thr1011 (2.56), Glu880 (2.43), Glu880 (2.80), Phe1143 (2.83), Tyr1141 (2.82), Tyr1141(2.75)
D-Glucosamine (3)	-6,17	Pro1013, Phe1143, Thr1011, Asn1149, Gly1140, Leu1139, Phe884	Glu880 (3.23), Glu880 (2.62), Tyr1141 (2.79), Tyr1141 (2.89), Pro1150 (3.01), Pro1150 (2.64)
		**ADA**	
Alliin (0)	-4,59	Trp113, Leu58, Leu102, Asp15, Tyr98, His13, Phe61	Ser99 (2.87), Arg97 (2.94)
Alliin (9)	-5,43	Thr265, Phe196, Asp292, Asp291, Ala179, Phe57, Leu54, Ser261	His13 (2.96), His234 (2.90), His11 (3.15), His210 (2.85), Gly180 (2.75), Gly180 (3.29)

Pyridoxamine (0)	-6,66	Cys149, Ser99, Arg97, Tyr98, Ala179, Phe61, Asp292, His13	Asp15 (2.68), Asp15 (2.72), His210 (2.83)
Pyridoxamine (8)	-7,26	Ser99, Tyr98, Leu103, Leu102, His13	Leu14 (2.79), Arg97 (2.76), Asp15 (2.67)
(*E*)-Ajoene (0)	-5,21	Ala169, Glu213, Asp291, Asp292, His13, Phe57, Asp15, Leu103, Tyr98, Ser99, Arg97	Gly180 (2.96), His210 (3.09)
(*E*)-Ajoene (5)	-5,74	Asp292, His13, Phe57, Asp15, Leu103, Leu102, Tyr98, Arg97, Cys149, Phe61, Leu14	Ser99 (2.66)
Pyridoxal (0)	-5,53	Leu102, Leu103, Leu14, Arg97, His13, Met151, Tyr98	Ser99 (2.74), Asp15 (2.80), Asp15 (3.04)
Pyridoxal (8)	-6,04	Leu102, Leu103, His13, Tyr98, Met65, Cys149, Asp15	Ser99 (2.59), Leu14 (2.71), Arg97 (2.66)
D-Glucosamine (0)	-6,30	Ala179, Leu54, Phe57, Phe296, His13, Glu213, Thr265	Gly180 (2.71), His210 (2.95), His234 (2.84), His11 (3.07), Asp291 (2.69)
D-Glucosamine (2)	-6,80	Ala179, Leu54, His13,	Gly180 (2.94), His210 (2.89), His234 (2.77), His11 (3.15), Asp291 (2.69), Glu213 (2.40), Glu213 (3.07)
**GDA**			
Pyridoxamine (0)	-5,43	Asn166, Thr168, Ser209 Leu165	Leu208 (3.00), Asp167 (2.42), Asp167 (3.24)
Pyridoxamine (5)	-5,95	Asn166, Thr168, Ser209 Leu165	Lei208 (2.61), Asp167 (2.48), Asp167 (2.69)
D-Glucosamine (0)	-5,07	Leu92, Phe206, Asn166, Leu165, Ser209	Thr168 (2.88), Asp167 (2.83), Asp167 (3.31), Asp167 (2.72), Leu208 (2.85)
D-Glucosamine (4)	-5,86	Phe206, Ser209, Leu246	Thr168 (3.33), Thr168 (2.90), Asp167 (2.55), Leu208 (2.80), Leu208 (2.93), Leu165 (3.18), Asn166 (2.97)

**PNP**			
Pyridoxamine (0)	-6,33	Met217, Phe198, Glu199, Ala115	Gly116 (2.72), Val215 (2.75), Ser197 (2.60), Pro196 (2.59)
**NT5C2-1497**			
4-Guanidinobutyric acid (0)	-5,10	Met453, Asp457	Lys359 (2.90), Asn36 (2.51), Arg37 (2.92)
4-Guanidinobutyric acid (5)	-5,49	Met453, Ala456, Arg454	Lys359 (2.83), Asn36 (2.56), Arg37 (3.19), Asp457 (2.79)
Pyridoxamine (0)	-4,31	Asp143	Thr145 (3.30), Thr145 (2.87), Glu146 (3.03), Asp144 (2.76)
Pyridoxamine (9)	-5,23	Glu146, Asp143, Gln141	Asp144 (2.59), Thr145 (3.07), Thr145 (2.80), Thr145 (2.83)
**NT5C2-1498**			
Glucosamine (0)	-4,19	Arg132	Pro129 (2.90), Glu130 (2.66), Glu130 (2.93), Glu133 (2.60)
Glucosamine (4)	-5,18	Arg132	Pro129 (2.85), Glu130 (2.51), Glu130 (2.93), Glu133 (2.69), Glu133 (2.51)
